# Interdependency of Brassinosteroid and Auxin Signaling in *Arabidopsis*

**DOI:** 10.1371/journal.pbio.0020258

**Published:** 2004-08-24

**Authors:** Jennifer L Nemhauser, Todd C Mockler, Joanne Chory

**Affiliations:** **1**Plant Biology Laboratory, Salk Institute for Biological StudiesLa Jolla, California, United States of America; **2**Howard Hughes Medical Institute, La JollaCaliforniaUnited States of America

## Abstract

How growth regulators provoke context-specific signals is a fundamental question in developmental biology. In plants, both auxin and brassinosteroids (BRs) promote cell expansion, and it was thought that they activated this process through independent mechanisms. In this work, we describe a shared auxin:BR pathway required for seedling growth. Genetic, physiological, and genomic analyses demonstrate that response from one pathway requires the function of the other, and that this interdependence does not act at the level of hormone biosynthetic control. Increased auxin levels saturate the BR-stimulated growth response and greatly reduce BR effects on gene expression. Integration of these two pathways is downstream from BES1 and Aux/IAA proteins, the last known regulatory factors acting downstream of each hormone, and is likely to occur directly on the promoters of auxin:BR target genes. We have developed a new approach to identify potential regulatory elements acting in each hormone pathway, as well as in the shared auxin:BR pathway. We show that one element highly overrepresented in the promoters of auxin- and BR-induced genes is responsive to both hormones and requires BR biosynthesis for normal expression. This work fundamentally alters our view of BR and auxin signaling and describes a powerful new approach to identify regulatory elements required for response to specific stimuli.

## Abstract 2

Although distinct sets of growth regulators - auxin and brassinosteroids - are required for cell expansion; rather than being independent signals, the response from each pathway requires the other

## Introduction

The continuous shaping of plant form is a marvel of signal integration. In early seedling development this is particularly clear, as environmental cues, such as light, profoundly alter the innate morphogenetic program. How diverse pathways merge to determine a discrete cellular growth response is largely unknown. Auxin, the first plant hormone identified, has been implicated in patterning or growth of virtually every plant tissue from earliest embryo to developing fruit (Liscum and Reed 2002). Brassinosteroids (BRs), the polyhydroxylated steroid hormones of plants, have been linked to many of these same processes, including photomorphogenesis (Clouse 2002). The nature of the relationship between these hormones has remained largely undefined.

Many factors in the signal transduction pathways operating downstream from BRs and auxin have been identified. Brassinosteroid Insensitive-1 (BRI1), a plasma-membrane-localized receptor serine/threonine kinase, is essential for BR perception and accounts for most BR-binding activity in *Arabidopsis* (Wang et al. 2001). A Shaggy/GSK3-type kinase, Brassinosteroid Insensitive-2 (BIN2), acts as a negative regulator of the pathway downstream of BRI1 action (Li and Nam 2002). When BR levels are low, proteins in the BES1/BZR1 family are hyperphosphorylated by BIN2 and targeted for degradation by the proteasome (He et al. 2002; Yin et al. 2002a). Upon BR perception, BIN2 is inactivated by an unknown mechanism which allows hypophosphorylated BES1/BZR1 proteins to accumulate in the nucleus, where they presumably provoke changes in gene expression (He et al. 2002; Yin et al. 2002a).

In contrast to BRs, no auxin receptor has been identified. However, exposure to auxin is known to promote rapid turnover of nuclear Aux/IAA proteins by ubiquitin-mediated targeting to the 26S proteasome (Gray et al. 2001). Aux/IAAs are direct negative regulators of the Auxin Response Factor (ARF) family of transcription factors and contain four highly conserved domains numbered I to IV (Abel et al. 1995). Domains III and IV are also found in most ARFs and facilitate dimerization within and between members of both families (Kim et al. 1997; Ulmasov et al. 1997b). ARF proteins bind to a conserved auxin-responsive element (AuxRE) found upstream of many auxin-regulated genes (Ulmasov et al. 1999).

Previous studies have suggested that auxin and BRs may have a particularly close relationship among plant hormones. In a variety of bioassays representing diverse species, BRs have been shown to synergistically promote cell elongation when supplied with auxin (Mandava 1988). Clouse and colleagues examined the effect of the two hormones on gene transcription more than a decade ago, and found that while BRs could activate the expression of some auxin-responsive genes, others appeared to be auxin specific (Clouse et al. 1992; Zurek et al. 1994). They also noted that detectable BR effects required much longer treatments compared with the extremely rapid effects of auxin, and concluded that BR-mediated cell elongation effects were likely independent from the auxin signal transduction pathway. Microarray experiments, assaying approximately one-third of the *Arabidopsis* genome, rekindled interest in the interaction between auxin and BRs, as it was found that a significant percentage of the BR genomic response comprised genes annotated as auxin responsive (Goda et al. 2002; Mussig et al. 2002; Yin et al. 2002a). Recent work from Nakamura and colleagues has shown that three genes—*IAA5, IAA19,* and *SAUR-AC1*—are induced by both auxin and BRs and that induction requires BR biosynthesis (Nakamura et al. 2003a, 2003b).

In this work, genetic, physiological, and genomic approaches were used to dissect the relationship between auxin and BRs in seedling growth. Together these techniques demonstrated that the relationship between these hormones is far more deeply intertwined than previously suspected. Auxin and BR effects on cell elongation were found to be interdependent, and this physiological interdependency was mirrored at the transcriptional level. In addition, growth and transcriptional effects of exogenous BR treatment could be largely superceded by overstimulation of the auxin pathway. Several lines of evidence suggested that auxin:BR synergism did not depend upon biosynthetic regulation of hormone levels; rather, the two response pathways are likely to converge at the promoters of shared target genes. New computational approaches detected a number of known transcription factor–binding motifs enriched in promoters induced by both hormones, as well as motifs which are overrepresented in promoters activated specifically by auxin or BRs. This multifaceted approach elucidates the mechanism of action of both auxin and BRs in cell expansion, and serves as a model for interrogating complex signaling networks.

## Results

### Auxin and BRs Interact Synergistically to Promote Hypocotyl Elongation

Early studies of BR effects in a variety of bioassays suggested that there was a synergistic interaction between auxin and BRs (Mandava 1988). We confirmed and extended these studies to the reference plant *Arabidopsis thaliana,* using hypocotyl (primary stem) length as a quantitative measure of growth. Both hormones are known to induce cell elongation, and exogenous BR treatment has been shown to increase hypocotyl length (Nemhauser et al. 2003). In contrast, addition of auxin to media has only modest effects on seedling hypocotyl elongation, likely as a result of inefficient acropetal transport from root to shoot (Gray et al. 1998). However, increased temperature has been demonstrated previously to be an effective method of altering auxin levels in the shoot and leads to robust increases in hypocotyl length (Gray et al. 1998; Zhao et al. 2002).

**Figure 1:**
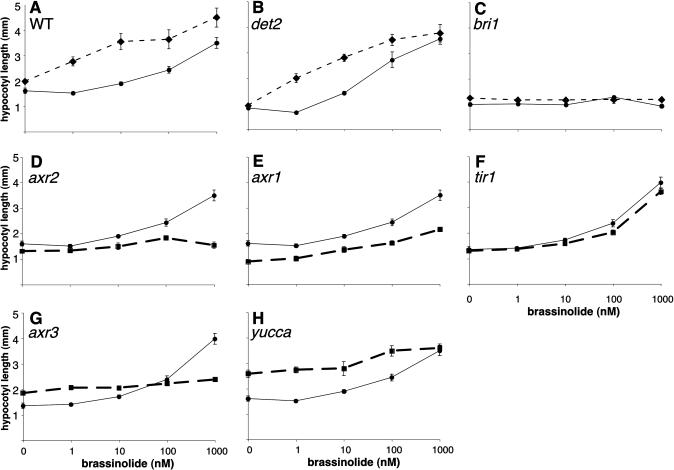
BR and Auxin Pathways Are Interdependent, as Measured by Hypocotyl Elongation (A) Mild temperature elevation causes elongation of the hypocotyl and BR hypersensitivity in WT plants. Columbia ecotype is shown but results are similar for Wassilewskija. Hypocotyls of 3-d-old plants grown at either 26 °C (diamonds, dashed line) or 22 °C (circles, solid line) were measured. (B) *det2-1* plants are defective in BR biosynthesis and are also insensitive to the temperature increase. As the *det2* deficiency is rescued by exogenous BL, temperature sensitivity is restored. (C) Plants with the weak *bri1-5* mutation are insensitive both to temperature and exogenous BR. (D–H) BR response depends upon auxin response. WT is shown in circles with a solid thin line and mutants are shown in squares with a thick dashed line. Known auxin response mutants *axr2-1* (D), *axr1-12* (E), *tir1-1* (F), and *axr3-1* (G) have decreased BR response. (F) *tir1* has no hypocotyl elongation phenotype in the absence of exogenous hormone treatment and only very modest effects on BR sensitivity. Response is significantly reduced in *tir1* mutants at 100 nM BL, as measured by Student's t-test (*p* = 0.03, using Bonferroni adjustment for multiple tests; Hochberg 1988). (H) *yucca* plants, which overproduce auxin, also show reduced BR response. Error bars represent standard error. Data in (F) and (G) were collected in a separate experiment from other panels, resulting in small differences in the values for WT hypocotyl length.

In our conditions, hypocotyls of plants grown at 29 °C were approximately 1.8 times longer than those of plants grown at 22 °C, consistent with what has been observed by others (Gray et al. 1998; Zhao et al. 2002). When exogenous brassinolide (BL), the most biologically active BR, was applied, hypocotyls of plants grown at elevated temperature exhibited a “kinked” morphology and agravitropic growth, typical of saturating BL conditions (data not shown). In order to examine the relationship between auxin and BRs, it was necessary to find conditions where auxin levels were increased but at subsaturating levels for the hypocotyl growth-promoting response. Plants grown at 26 °C versus 22 °C showed measurable increases in both hypocotyl elongation and levels of auxin intermediates (Zhao et al. 2002). Using these conditions, it was possible to observe that plants grown at higher temperatures were more sensitive to exogenous BR treatment, both in threshold levels for response as well as in terms of absolute growth (Figure 1A).

**Figure 2:**
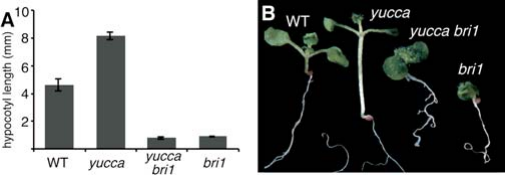
Enhanced Hypocotyl Elongation of *yucca* Mutants Requires Functional BRI1 (A) Average hypocotyl lengths of 3-d-old plants. Error bars represent standard error. (B) Ten-day-old WT, *yucca, yucca bri1-116,* and *bri-116* seedlings.

BR- and auxin-mediated growth promotion required both pathways to be intact. As has been shown previously, hypocotyls of *det2* mutants defective in BR biosynthesis (Li et al. 1996) fail to elongate with increased temperature (Gray et al. 1998; Zhao et al. 2002). Importantly, the hypersensitivity of *det2* plants to exogenous BR was enhanced by increased temperature, suggesting that these two responses are tightly linked (Figure 1B). Weak *bri1* mutants were also unresponsive to temperature, suggesting that auxin response was dependent on a functional BR signal transduction pathway (Figure 1C). The dramatic growth enhancement caused by overproduction of auxin in the *yucca* mutant (Zhao et al. 2001) requires functional *BRI1,* as *yucca bri1* mutants are dwarfs (Figure 2). Conversely, BR response was dependent on a functional auxin signal transduction pathway as *axr1* (Lincoln et al. 1990) and *axr2* (Timpte et al. 1994) mutants with reduced auxin response showed significantly reduced sensitivity to BR treatment (see Figure 1D and 1E). The degree of BR insensitivity is correlated with the level of reduced auxin responsiveness, as *tir1* mutants, which show only subtle phenotypes in the absence of exogenous auxin (Ruegger et al. 1998), exhibited only a modest reduction in BR response (see Figure 1F). *axr3* mutants, which in many assays display a constitutive auxin response (Leyser et al. 1996), were insensitive to BRs (see Figure 1G). This suggests that the BR insensitivity observed in *axr1* and *axr2* mutants is not simply a block in cell elongation, and that regulated turnover of Aux/IAA proteins, such as those encoded by *AXR2* and *AXR3,* is required for normal BR response. *yucca* mutants were also largely insensitive to exogenous BR and appeared saturated for the BR response (see Figure 1H).

### Auxin and BR Transcriptional Responses Substantially Overlap

**Figure 3:**
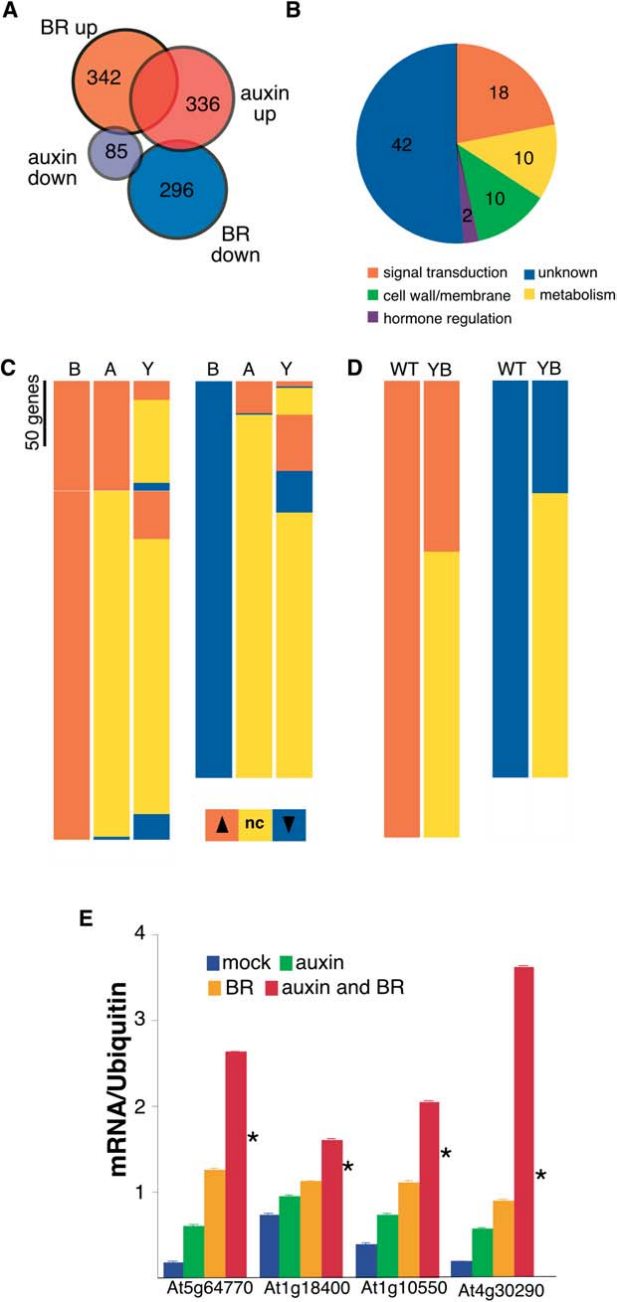
BR and Auxin Have Shared Genomic Effects (A) Venn diagram showing relative proportion of BR- and auxin-responsive genes and the degree of overlap. (B) Functional categories of BR–auxin shared genes reveal a potential growth signature. (C and D) Effects of auxin on BR-regulated gene expression. Transcripts which show elevated levels are shown in orange, those with decreased levels are shown in blue, and those transcripts whose levels are not changed are shown in yellow. (C) Relative ratios were derived from the following comparisons (from left to right): BR versus mock treatment (WT plants; B), auxin versus mock treatment (WT plants; A), and *yucca* versus WT (Y). The three columns to the left are BR-upregulated genes and the three columns to the right are BR-downregulated genes. Among the BR-upregulated genes, there are a large number that are also induced by auxin treatment or in a *yucca* background. Few BR-repressed genes are repressed by auxin. nc, no change. (D) Effect of BR treatment in *yucca* background. Relative ratios represent BR versus mock treatment in WT plants (WT) or in *yucca* mutants (YB). Approximately two-thirds of BR-regulated genes were not affected by BR treatment of *yucca* plants. (E) Quantitative PCR shows that shared target genes are synergistically induced when treated with both auxin and BRs. At5g64770 encodes a protein with unknown function. At1g18400 encodes BEE1, a bHLH-containing protein known to be required for the BR response (Friedrichsen et al. 2002). At1g10550 and At4g30290 are putative endoxyloglucan transferases. Asterisks indicate response under an additive model.

Previous studies have identified several auxin-responsive genes that are also regulated by BRs (Goda et al. 2002; Mussig et al. 2002; Yin et al. 2002a; Nakamura et al. 2003a, 2003b). To comprehensively compare the genomic effects of treatment with each hormone, Affymetrix oligonucleotide microarrays, representing approximately 22,000 genes, were hybridized with probes from two biological replicates following mock or BR treatment. Linear models were used to identify 342 transcripts whose levels were increased following BR treatment (Figure 3A; Tables S1 and S3). The levels of 296 transcripts were decreased in the same treatment (Figure 3A; Tables S2 and S4). Comparison with newly analyzed data from a similar experiment using auxin-treated seedlings (Zhao et al. 2003) showed that nearly a quarter of genes upregulated by either auxin or BR treatment were regulated by both hormones (Figure 3A and 3C; Tables S1–S6). This is a much larger overlap than that reported in the recent study by Goda and colleagues (2004), likely reflecting substantial differences in experimental design and analysis methods, including the use of different microarrays. In addition, at least 75% of the genes identified as BR inducible were late responders (only observed after 12 or 24 h of BR treatment) and therefore were not included in the analysis described here.

As much of the auxin response is transient, *yucca* plants which continuously experience high levels of auxin have a different profile of altered transcript levels than plants exposed to exogenous auxin for a short time period (Zhao et al. 2002). To produce a more complete list of auxin-responsive genes, RNA from *yucca* seedlings was isolated and used to probe additional microarrays. More than 20% of all BR-upregulated genes were also differentially regulated in a *yucca* background (Figure 3C; Tables S1 and S2). In combination, 40% of the BR-upregulated genes were altered either by auxin treatment or in *yucca* mutants (see Table S1). Members of all known auxin-responsive gene families were identified, as has been seen in previous microarray experiments representing a smaller fraction of the genome (Goda et al. 2002, 2004; Mussig et al. 2002; Yin et al. 2002a).

While auxin treatment had no effect on ARF gene expression, transcripts of *ARF4* (At4g30080) and *ARF8* (At5g37020) were negatively regulated by BR treatment (see Tables S2 and S4). This is the first evidence of transcriptional regulation of ARF genes. In addition, BRs repressed the expression of several auxin transport–related transcripts, including *PIN3* (At1g70940), *PIN4* (At2g01420), *PIN7* (At1g23080), and an *AUX1*-like gene (At1g77690). Auxin induced the expression of *BRI1* and a close paralog, *BRL3* (At3g13380), and repressed the expression of another *BRI1*-like gene, *VH1/ BRL2* (At2g01950) (Clay and Nelson 2002; Yin et al. 2002b). It is possible that the genes identified here as auxin and BR responsive may represent a common growth signature regulated by many factors during seedling development. The majority of these genes do not have known functions; however, many of the rest are known or predicted to be involved in cell expansion, metabolism, and signal transduction (Figure 3B).

### Integration between Auxin and BR Signals Occurs in the Nucleus

**Figure 4:**
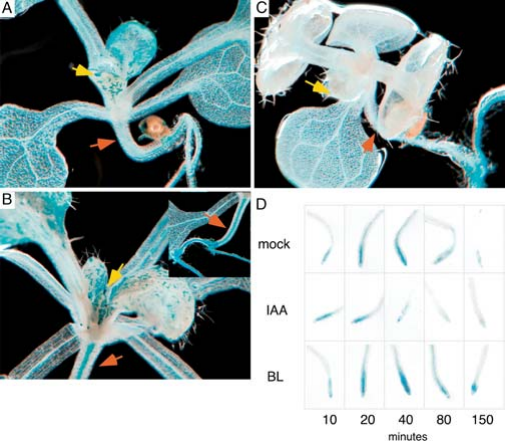
Endogenous BR Levels Affect Expression of an Auxin-Responsive Reporter but Do Not Induce Aux/IAA Protein Turnover (A) WT, (B) *det2*, and (C) *DW4FOX* plants carrying the DR5::GUS transgene. (A) GUS staining is particularly strong in young leaves (yellow arrow). (B) *det2* plants show no GUS staining in aerial tissues. (C) *DWF4OX* plants show increased intensity of staining, particularly at the tips of emerging leaves (yellow arrow) and in the hypocotyl (orange arrows). Inset shows hypocotyl-root junction. (D) Aux/IAA stability does not appear to be affected by treatment with BRs. Plants carrying a heat shock–inducible fusion of the N-terminal portion of AXR3 and GUS reporter were subjected to 2 h at 37 °C and then treated with mock or hormone treatments for the time periods listed.

Many plant hormones directly regulate the levels of other hormones (Alonso and Ecker 2001). This complicates analysis of cross-talk, which is defined by shared signal transduction components. The interdependency between auxin and BRs does not function primarily through regulation of hormone levels. Auxin does not induce BR biosynthesis. *det2* plants, which are hypersensitive to exogenous BR treatment, were insensitive to growth at elevated temperature (see Figure 1B). Auxin treatment does not affect the subcellular localization of BES1 (Yin et al. 2002a), and growth at elevated temperature does not alter BES1 levels or phosphorylation state (unpublished data). Conversely, BRs do not regulate auxin biosynthesis. Nakamura and colleagues (2003a) reported that *det2* mutants make at least normal amounts of auxin and that BR treatments do not alter auxin levels. It was recently reported that the stability of an IAA1:luciferase fusion protein was unchanged following BR treatment, though the data were not shown (Zenser et al. 2003). Here, we used a heat shock–inducible β-glucuronidase (GUS) reporter fused to the N-terminal portion of AXR3 described by Gray and colleagues (2001). This construct was rapidly turned over in the presence of auxin but showed no change in stability following BR treatment (Figure 4D).

Together, these results suggested that the interaction between the auxin and BR pathways was likely at the promoters of shared target genes. To test whether the auxin:BR synergism was detectable at the level of gene transcription, transcript levels from four genes identified in the microarray studies were quantified in plants exposed to exogenous treatment of either hormone or both in combination (see Figure 3E). In all cases, levels of these transcripts were regulated nonadditively in the presence of both hormones. If, as suggested by these results, BR and auxin response pathways converge at the level of gene activation, we reasoned that *yucca* plants, which are largely insensitive to BR for growth promotion, might also show a reduced BR genomic response. RNA was isolated from *yucca* plants treated with BR and used to probe additional microarrays. Approximately two-thirds of genes showing BR responsiveness in wild-type (WT) plants were no longer affected by BR treatment in a *yucca* background (see Figure 3D; Tables S1 and S2). This result strongly suggests that auxin and BR treatment affect transcription of these target genes by a common mechanism.

### Promoters of Coordinately Regulated Genes Share Regulatory Motifs

Computational analysis of coordinately regulated genes is an emerging tool for dissecting regulatory networks (e.g., DeRisi et al. 1997; Harmer et al. 2000; Tullai et al. 2004). To identify potential regulatory elements acting in these pathways, a list of all genes regulated by either auxin or BR was generated, and 500 bp upstream of each gene were identified. These promoters were split into three groups: those with increased transcript levels following treatment with BR only (B group; *n* = 258), those with increased transcript levels following auxin treatment only (A group; *n* = 254), and those genes whose transcripts were induced following treatment with either hormone (AB group; *n* = 82). Known plant promoter elements and their annotations were downloaded from PLACE (Higo et al. 1999) and used to screen each promoter list. The expected number of occurrences of each PLACE motif was estimated using 1,000 sets of *n* promoters randomly sampled from the genome, where *n* is equal to the number of promoters in each group (A, B, or AB). This approach offers a significant advantage over other background models used to assess enrichment. Permuted distributions reflect real expected frequencies and do not rely on assumptions about genome architecture. In addition, the normal distribution of site frequencies observed with large numbers of permutations allows for the use of powerful parametric statistical methods. Moreover, the ease of filtering based on relative probabilities makes this approach ideally suited to comparisons of promoters regulated in different conditions. In this study, matches were considered significant if a motif was overrepresented in a given set (*p* < 0.1) and present in at least 10% of group promoters. This analysis identified several motifs specifically enriched in a given group (Table 1), as well as several motifs found to be enriched in multiple groups (Table 2).

**Table 1:**
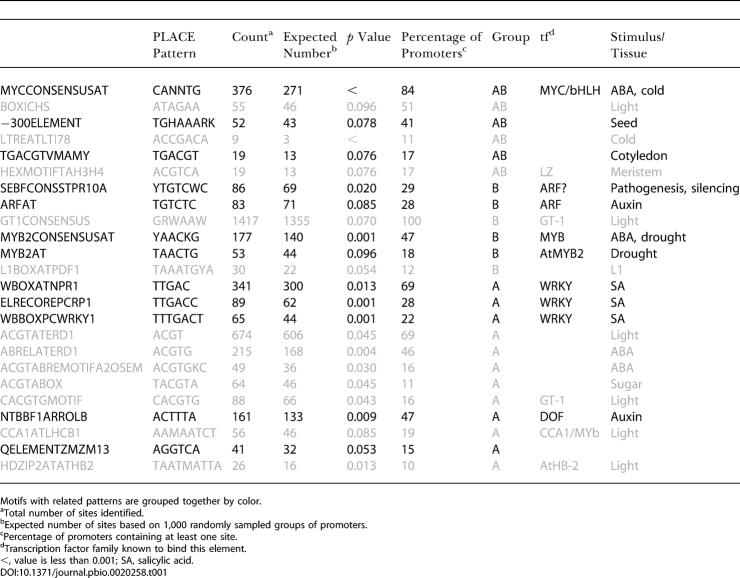
PLACE Motifs Enriched Specifically in AB, A, or B Promoters

Motifs with related patterns are grouped together by color

^a^Total number of sites identified

^b^Expected number of sites based on 1,000 randomly sampled groups of promoters

^c^Percentage of promoters containing at least one site

^d^Transcription factor family known to bind this element

<, value is less than 0.001; SA, salicylic acid

DOI:10.1371/journal.pbio.0020258.t001

**Table 2:**
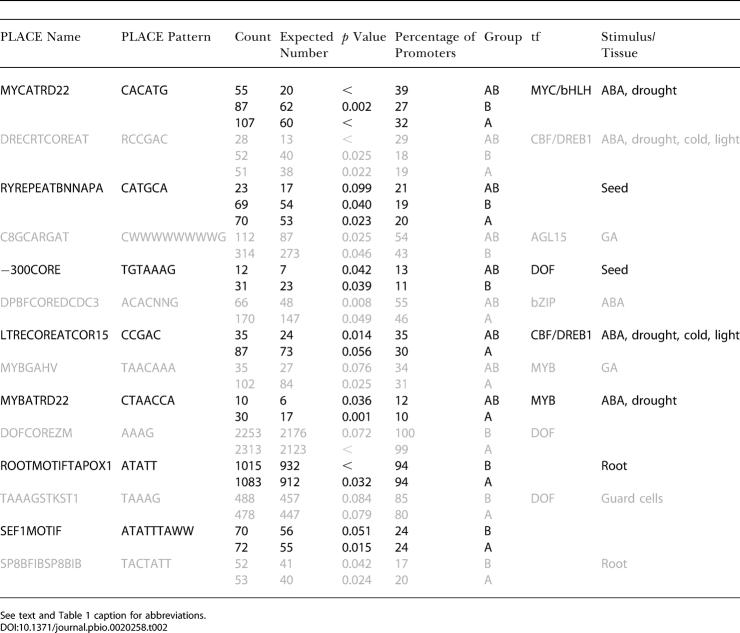
PLACE Motifs Enriched in Promoters of Multiple Groups

See text and Table 1 caption for abbreviations

DOI:10.1371/journal.pbio.0020258.t002

One of the sequences enriched in the B group was TGTCTC, previously identified as an auxin-responsive element (Ulmasov et al. 1995) termed ARFAT in the PLACE database. Surprisingly, this sequence was not significantly enriched in the A set (*p* = 0.78). However, the A, B, and AB groups showed significant enrichment of the core ARF-binding element TGTC in their promoters, perhaps reflecting some sequence divergence between *Arabidopsis* and soybean, where the element was first identified. A well-characterized synthetic element containing the ARFAT called DR5 (Ulmasov et al. 1997a) could be used to test the BR responsiveness of this element and was therefore introduced into plants with altered BR levels. In *det2* plants with lower endogenous levels of BRs (Li et al. 1996), DR5 expression was greatly reduced, particularly in the shoot (Figure 4A versus 4C). Conversely, in plants with increased levels of BRs caused by overexpressing a BR biosynthetic gene, *DWF4* (Wang et al. 2001), DR5 expression was increased (Figure 4A versus 4B). DR5 expression was also increased following transient BR treatment of WT plants carrying the DR5 reporter (unpublished data). Nakamura and colleagues (2003a) also recently demonstrated the BR inducibility of DR5::GUS and found no change in endogenous IAA levels following BR treatment, providing further evidence that BR transcriptional effects are direct. These data strongly suggest that the ARF-binding element requires both hormones for proper expression and should be considered a Brassinosteroid-Auxin Response Element. This finding raises questions about the utility of the DR5 element as a reporter of auxin response, as it likely reflects regions of regulatory overlap between the two pathways.

Consensus binding sites for several families of transcription factors were identified as enriched in the AB set (see Table 1). The presence of a MYC consensus site in more than 80% of AB promoters was quite striking, especially in light of the BR and auxin inducibility of the bHLH-containing *Brassinosteroid Enhanced Expression 1* (*BEE1;* At1g18400) gene, which is known to function in BR response (Friedrichsen et al. 2002). Many of the other AB consensus motif matches were implicated in regulation by light or abscisic acid (ABA), both of which have been linked previously to BR-mediated growth response by physiology and genetics (Nemhauser and Chory 2002). For the B set, there was widespread occurrence of a GT-1 consensus binding motif, as well as evidence for a MYB-binding site distinct from that found in the A set. Identification of several elements specific for the A set, including those known to bind WRKY-family members, suggests attractive targets for designing new reporters which may not be BR dependent. Several instances of light-regulated motifs are intriguing given the strong evidence for a close relationship between auxin and light responses (Tian and Reed 2001).

Several of the promoter elements identified in the A, B, and AB promoters were found as multiple copies within promoters, including the core ARF-binding element TGTC. Recent studies have suggested that ARF dimerization is not required for activation of ARFAT-mediated transcription (Tiwari et al. 2003). Interestingly, a scan of AB promoters revealed that nearly half of all AB promoters contain at least one instance of multiple copies of the core TGTC element within a 50-bp window. Clustering of TGTC sites was also seen in the A set (42% of promoters contain at least one pair of sites within 50 bp) and somewhat less frequently in the B set (33%). This finding suggests that interactions between ARFs may be important for hormone responsiveness of natural promoters, in addition to enhancing auxin inducibility of synthetic multimerized ARFATs. As specific binding factors are not known for most of the other elements identified, exact nucleotides required for factor binding are not known. Therefore, this analysis is likely a conservative estimate for the number of true transcription factor–binding sites present in each promoter.

## Discussion

With the notable exception of auxin, most plant hormones are produced and perceived throughout the plant body. Modulation of hormone response stems from regulation of hormone levels and/or signal transduction components, as well as from interactions with other signaling pathways. There are many examples of cross-talk between hormones in plant biology. In addition to auxin and BRs, gibberellins (GAs), ethylene, ABA, and cytokinin have all been shown to affect hypocotyl elongation (reviewed in Nemhauser and Chory 2002). As mentioned previously, some of these hormones interact through biosynthetic regulation. For example, auxin, ABA, and cytokinin stimulate ethylene biosynthesis, particularly when supplied at high levels (Yang and Hoffman 1984; Vogel et al. 1998; Ghassemian et al. 2000). Physiological and genetic evidence suggests that auxin, GAs, and ethylene promote hypocotyl growth by largely independent means (Gray et al. 1998; Collett et al. 2000). Similarly, BRs and GAs interact additively in most cell elongation bioassays (Mandava et al. 1981), and analysis of *bri1* mutants suggests that the two hormones independently and antagonistically regulate transcription of some target genes (Bouquin et al. 2001). In contrast, auxin and BRs interact synergistically and interdependently to promote hypocotyl cell elongation, making their relationship unique among plant growth regulators.

The nature of hormone interactions may be tissue specific. A recent study demonstrated that auxin acts primarily through GAs to promote root elongation, and proposed that the DELLA family of negative regulators was a point of convergence between the two pathways (Fu and Harberd 2003). One possible complication for this interpretation is that auxin is required for normal GA biosynthesis in pea (Ross et al. 2000) and thus, the effects of auxin on DELLA protein stability may be indirect. We have preliminary evidence that interactions between auxin and BRs may be different in aerial tissues than in roots. While auxin and BRs promote hypocotyl elongation, the hormones have opposite effects on root hair growth (J. L. Nemhauser and J. Chory, unpublished data). In addition, reduced BR levels or response may actually increase auxin effects on root pericycle proliferation (J. L. Nemhauser, N. Geldner, and J. Chory, unpublished data).

While auxin and BRs stimulate elongation of the hypocotyl, light antagonizes this effect. The AB genes induced by auxin and BRs may be targets for repression by the light response. Plants with reduced BR levels or response show a light-grown phenotype even when grown in the dark, including a short hypocotyl, expansion of cotyledons, and production of leaves. Many mutants with stabilized Aux/IAA proteins also show this deetiolated phenotype (Tian and Reed 2001). Levels of BRs may be light regulated (Kang et al. 2001), and response to BRs is affected by light quality and intensity (Nemhauser et al. 2003). Interestingly, two photoreceptors, PHOT1 (At3g45780) and Phytochrome E (At4g18130), are both downregulated by BRs. Two potential negative regulators of the light response, PKS1-like (At5g04190) and DRT100 (At3g12610), are upregulated by both auxin and BRs. Differential regulation of target genes by auxin, BRs, and light may allow fine-tuning of the photomorphogenetic response.

### Bioinformatic Analysis of Signaling Networks

The regulation of gene expression in eukaryotes is complex and is largely mediated by multiple transcription factors that bind within regulatory regions upstream of the coding sequence. In the simplest model, coexpressed genes exhibit similar expression characteristics because they are regulated by the same transcription factors. A number of algorithms have been developed to identify potential regulatory motifs overrepresented in the promoter sequences of coregulated genes (reviewed in Rombauts et al. 2003). Each algorithm requires a background model to calculate the expected frequency for each motif. The simplest background model estimates the expected frequency for a given motif based on the single nucleotide composition of the analyzed sequences (Bailey and Elkan 1995; Roth et al. 1998). Improvements on these methods use so-called higher-order models based on Markov chain statistics (Thijs et al. 2001, 2002; Marchal et al. 2003), building the background model by estimating the probability at each nucleotide position based on the previous bases in the sequence. Other approaches include enumerative methods that generate background models based on whole-genome motif counts from noncoding intergenic (van Helden et al. 1998) or randomly sampled (Marino-Ramirez et al. 2004) genomic sequences.

Because biological sequences are inherently nonrandom, we chose another approach to build our background model. For each motif under consideration, we modeled the expected frequency distribution by randomly sampling sets of promoter sequences from among all the genes represented on the microarrays used in our study. Therefore, we could directly estimate the statistical significance for each motif from its Z score, which is the number of standard deviations by which the observed frequency exceeds the expected frequency based on the distribution observed in the permutation sampling. In contrast to other methods, our approach uses a background model based on a real distribution of motif counts derived from annotated promoter sequences, rather than estimating expected word frequencies from simulated or randomly selected genomic sequences or from models based on distribution functions. Thus, given any set of *Arabidopsis* genes clustered on the basis of similar expression, we could easily identify overrepresented known transcription factor–binding motifs or overrepresented novel presumptive promoter elements. For example, new experiments assaying genomic effects of different hormone treatments or environmental conditions could be used to define finer groupings of coregulated genes and could be readily integrated into our current analysis.

### A Model for Auxin:BR Synergy

**Figure 5:**
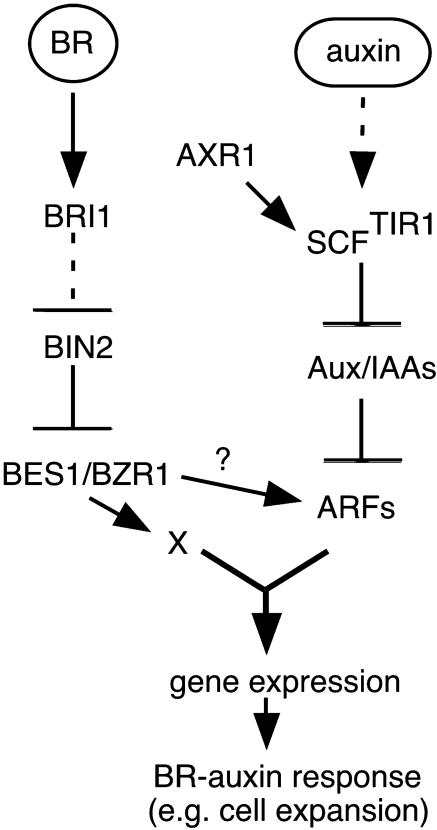
A Model of BR–Auxin Interaction Auxin and BR signals are likely integrated on promoters of shared target genes. The node(s) of intersection between auxin and BR pathways must be downstream of BES1 and Aux/IAAs and upstream of gene expression. One likely mechanism is via regulation of transcriptional complexes, such as those containing the ARFs.

Auxin:BR synergism results from convergence of the two response pathways on a common mechanism for promoting cell elongation. The integration of these hormone signals occurs very late in signal transduction, likely at the promoters of more than 80 genes whose expression is induced by short treatments with either hormone. Several known regulatory elements have been identified in these common target genes. The well-characterized auxin-response element ARFAT is one crucial node of intersection between the BR and auxin pathways, as it is BR responsive and requires BR synthesis for normal expression. More than 20 ARFs have been identified in the *Arabidopsis* genome (Liscum and Reed 2002). Many have been shown to bind the ARFAT motif and promote auxin-inducible gene expression (Ulmasov et al. 1997b; Tiwari et al. 2003). Stabilization of Aux/IAA proteins, such as AXR2 and AXR3, completely blocks BR growth responses. We propose a model where auxin and BR pathways converge on regulation of ARF transcription factors (Figure 5).

Cross-talk is a common feature of animal growth regulator pathways. For example, glucocorticoids synergistically enhance the effects of retinoic acid in mouse cells (Subramaniam et al. 2003). Upon ligand binding, the glucocorticoid receptor directly interacts with the homeodomain protein Pbx1 and activates transcription of Hoxb-1. In *Xenopus,* transcriptional activation of several genes, including *twin, siamois,* and *nodal-related-3,* requires stimulation of both TGFβ and WNT pathways. Similarly, two transcription factors, SOX10 and KROX20, have been recently reported to interdependently regulate expression of a neural crest–specific enhancer conserved among mouse, human, and chicken (Ghislain et al. 2003). This type of coregulation is also seen in plants. One example is the synergistic interaction between osmotic stress and ABA response, which is likely mediated by interaction between DREB and AREB transcription factors (Narusaka et al. 2003). In all of these cases, signal integration is achieved by formation of a complex containing transcription factors independently regulated by each pathway, often binding to composite regulatory elements. By integrating the inputs of multiple pathways, these mechanisms provide cellular or regional specificity for a given response.

ARFAT was originally identified as part of a composite element (Ulmasov et al. 1995). However, DR5 has been characterized as a multimerized simple response element (Ulmasov et al. 1997b) and can be activated by either auxin or BRs. So, unlike in the systems described above, auxin and BR signals likely converge on the same family of transcription factors. Such a relationship has recently been described for ethylene and jasmonate in plant defense responses (Lorenzo et al. 2003). Both ethylene and jasmonate pathways are required to induce expression of the transcription factor ERF1, which in turn regulates the expression of a number of defense-related genes. Neither auxin nor BRs have large effects on ARF transcription, and several AB targets are early-response genes not requiring de novo protein synthesis for activation (Friedrichsen et al. 2002; Liscum and Reed 2002). Auxin and BRs likely regulate ARF complex activity posttranslationally rather than through transcriptional regulation. Auxin is already known to modulate ARF activity by regulating the stability of the interacting Aux/IAA repressor proteins (Gray et al. 2001; Tiwari et al. 2003). BR perception could increase ARF activity by leading to modification of the ARFs themselves or through interactions with a BR-regulated transcriptional coactivator. The additional transcriptional regulation of some ARFs by BRs, together with auxin and BR effects on a number of Aux/IAA genes, could favor formation of particular transcriptional complexes promoting growth. Five genes encoding proteins with DNA-binding motifs were induced by both hormones, including members of the MYC, EREBP, and leucine zipper families. Higher-order interactions among several transcription factor complexes, perhaps directly involving members of the BES1/BZR1 family, could provide additional control of the shared auxin:BR response pathway.

A longstanding question in plant biology has been how a small number of hormones with overlapping functions can provoke a wide range of responses. Combinatorial control has long been suggested as one possible explanation (e.g., Singh 1998). The detailed analysis of BR and auxin pathways in this work suggests that hormone response is determined by the cellular milieu. Additional factors, including other hormones and environmental stimuli, can be incorporated into this model, leading ultimately to a detailed map of plant growth processes.

## Materials and Methods

### 

#### Hypocotyl measurements

Seeds were sterilized for 15 min in 70% ethanol, 0.01% Triton X-100, followed by 10 min of 95% ethanol. After sterilization, seeds were suspended in 0.1% low-melting-point agarose and spotted on plates containing 0.5× Murashige Minimal Organics Medium (Gibco-BRL, San Diego, California, United States), 0.8% phytagar (Gibco-BRL), and one of five concentrations of BL (0, 1, 10, 100, or 1,000 nM). Seeds on plates were then stratified in the dark at 4 °C for 2 d. Plants were grown in approximately 35 μmol m^−2^s^−1^ white light with a red:far-red light ratio near 1. Plate position was changed every 24 h to minimize position effect. Hypocotyl lengths were measured from 10 to 14 3-d-old seedlings. Seedlings were removed from one plate at a time and scanned between two transparencies on a flatbed scanner. NIH Image 1.62 was used to perform length measurements. All dose-response experiments were performed in duplicate. *bri1-5* is a weak allele in a Wassilewskija background. All other mutants used in this work are in a Columbia background.

#### GUS staining

GUS staining protocol was as described in Sessions et al. (1999). Induction of AXR3-NT-GUS lines was as described in Gray et al. (2001).

#### Microarray studies

Nine-day-old, light-grown *Arabidopsis* seedlings were immersed in 1 μM BL in 0.5× Murashige Minimal Organics Medium (Invitrogen, Carlsbad, California, United States) or medium alone for 2.5 h before they were harvested for total RNA preparation. Total RNA from the treated seedlings was used for preparing probes for the microarray experiments, which were carried out according to the protocols provided by the gene chip manufacturer Affymetrix (Santa Clara, California, United States). All experiments used two independent biological replicates. Details of the auxin experiment have been described previously (Zhao et al. 2003). Data analysis was performed in R (Ihaka and Gentleman 1996). Genes were normalized using rma in the Bioconductor affy package (http://www.bioconductor.org; Irizarry et al. 2003) and subsequently analyzed using linear models and Empirical Bayes analysis (limma package; Smyth 2004). To be considered differentially expressed, genes were required to have a false discovery rate adjusted *p* value of less than 10% and an empirical Bayes log odds of differential expression (B) greater than 0. Data are available at Gene Expression Omnibus; see Supporting Information for accession numbers.

#### Quantitative PCR

Plants were treated with hormones as above using treatments of either 1 μM BL, 1 μM indole-3-acetic-acid (auxin), both hormones, or a mock treatment. Total RNA was extracted using a Qiagen (Valencia, California, United States) RNAeasy kit and first-strand cDNA was synthesized using an Invitrogen Superscript First-Strand cDNA Synthesis kit. cDNAs were diluted 20-fold and combined with SYBR master mix (PE Biosystems, Wellesley, California, United States) for PCR. Primers were as follows: At5g64770 (5′-CTTCTCATACTCTTCATTTCCTCTCCTACT-3′, 5′-TTCTCGTAAGCTTCGTGCTTGA-3′), At1g18400 (5′-CTAGCGGCGTCTCCGATAAT-3′, 5′-AAGAACCTGTTTCAGTGGCAATAAC-3′), At1g10550 (5′-AAGCTTCCCGCTGGATTTG-3′, 5′-TTGATAAATAGAAAGCAACCACAACAC-3′), and At4g30290 (5′-TCCCTGGTAACTCTGCTGGAA-3′, 5′-CCGGAGATTTAAGATAGAATGTTGTGA-3′). At5g15400 (ubiquitin) was used to normalize all values (5′-TGCGCTGCCAGATAATACACTATT-3′, 5′-TGCTGCCCAACATCAGGTT-3′). PCR reactions were performed in triplicate and analyzed using an ABI PRISMA 7700. A standard curve was constructed for each primer using an equal mixture of all cDNAs.

#### Sequences for promoter analysis

We used a Perl script to extract the 500 bp of sequence preceding the 5′ end of each annotated transcription unit in the AGI pseudomolecules annotation (14-May-2003) downloaded from NCBI. These putative promoter sequences begin immediately upstream of the 5′ UTR for transcription units with an annotated 5′ UTR, and upstream of the annotated translational start for the remainder.

#### Promoter analysis and significance calculations

We analyzed putative promoter regions upstream of auxin- and BR-regulated genes to identify overrepresented promoter elements. One thousand surrogates of each promoter set were created by randomly shuffling the list of genes represented on the Affymetrix ATH1 arrays and then sampling *n* genes and extracting 500-bp promoter sequences for the sampled set of genes. Known plant promoter elements and their annotation were downloaded from PLACE (Higo et al. 1999). For each set of *n* promoters, the null distribution for each PLACE motif was modeled by counting the number of occurrences for each word within each of the 1,000 surrogate sets of *n* promoters. Using this approach we could then ask how well the observed frequency of a certain motif in a set of *n* promoters matched the frequency that would be expected for a random set of *n* promoters. We estimated the one-tailed *p* value for each motif based on the Z score of the difference of the actual word count of the promoter set (C_true_) minus the mean count from the 1,000 surrogates (C_surr_) relative to the SD from the 1,000 surrogates (SD_surr_) [i.e., Z = (C_true_ − C_surr_)/SD_surr_]. Thus for each motif the *p* value we calculated was the probability to the right of the observed count calculated on the null distribution derived from sampling promoters randomly from the genome. We considered a motif to be significantly overrepresented if this probability was less than 0.1. These calculations were implemented using Perl scripts and a relational database (MySQL).

## Supporting Information

Table S1 Fold Change of BL-Upregulated Genes following Exposure to BL or IAA (Auxin) Treatment Effects of increased auxin levels in the *yucca* mutant are shown as compared to WT and following BL treatments. The comparisons from left to right are WT BL- versus mock-treated, WT IAA- versus mock-treated, *yucca* mock-treated versus WT mock-treated, and *yucca* BL- versus mock-treated. nc, no change.
(160 KB XLS).

Table S2 Fold Change of BL-Downregulated Genes following Exposure to BL or IAA (Auxin) Treatment Effects of increased auxin levels in the *yucca* mutant are shown as compared to WT and following BL treatments. The comparisons from left to right are WT BL- versus mock-treated, WT IAA- versus mock-treated, *yucca* mock-treated versus WT mock-treated, and *yucca* BL- versus mock-treated. nc, no change.
(169 KB XLS).

Table S3 Normalized Values of BL-Upregulated Genes ave, average; se, standard error.
(64 KB XLS).

Table S4 Normalized Values of BL-Downregulated Genes ave, average; se, standard error.
(64 KB XLS).

Table S5 Values of IAA-Upregulated Genes ave, average; se, standard error.
(63 KB XLS).

Table S6 Normalized Values of IAA-Downregulated Genes ave, average; se, standard error.
(26 KB XLS).

### Accession Numbers

The Gene Expression Omnibus (http://www.ncbi.nlm.nih.gov/geo/) accession numbers for the genes and gene products discussed in this paper are WTBR1 (GSM13423), WTBR2 (GSM13424), WTmock1 (GSM13420), WTmock2 (GSM13421), wtzm1 (GSM13430), wtzm2 (GSM13432), wtzmIAA1 (GSM13433), wtzmIAA2 (GSM13434) and, *yucca*BR1 (GSM13428), *yucca*BR2 (GSM13429), *yucca*mock1 (GSM13426), and *yucca*mock2 (GSM13427).The associated experimental descriptions are available at accession numbers GSE862 (BR effects on WT and *yucca* seedlings) and GSE863 (auxin effects on seedlings).

## Abbreviations

ABA: abscisic acid
ARF: Auxin Response Factor
BL: brassinolide
GA: gibberellin
GUS: β-glucuronidase
WT: wild-type
